# Profile, outcomes, and predictors of mortality of abdomino-pelvic trauma patients in a tertiary ICU in Saudi Arabia

**DOI:** 10.1186/cc13228

**Published:** 2014-03-17

**Authors:** S Haddad, Z Youssef, S Azzam, A Aldawood, A Al-Zahrani, H Al-Zamel, H Tamim, A Deeb, Y Arabi

**Affiliations:** 1 King Abdulaziz Medical City, Riyadh, Saudi Arabia

## Introduction

Saudi Arabia (KSA) has the world's highest number of deaths from motor vehicle accidents (MVAs). Numerous trauma victims sustain abdomino-pelvic injuries which are associated with considerable morbidity and mortality. The purpose of this study is to describe the profile, outcomes and predictors of mortality of patients with abdomino-pelvic trauma admitted to the ICU in a tertiary care trauma center in Riyadh, KSA.

## Methods

This is a retrospective analysis of a prospectively collected ICU database. All consecutive patients older than 14 years with abdomino-pelvic trauma from March 1999 to June 2013 were included. The followings were extracted: demographics, injury severity, mechanism and type of injury, associated injuries, use of vasopressors and mechanical ventilation, and worse laboratory results in the first 24 hours. The primary outcome was hospital mortality. Secondary outcomes were ICU mortality, mechanical ventilation duration, need for tracheotomy, and ICU and hospital length of stay. We compared the profile, outcomes, and the predictors of mortality between survivors and nonsurvivors.

## Results

Of 9,974 trauma patients during the study period, 702 patients with abdomino-pelvic trauma were admitted to the ICU. The average age was 30.7 ± 14.4 years and the majority was male (89.5%). MVA was the most common cause of abdomino-pelvic trauma (86%). Pelvis (46.2%), liver (25.8%), and spleen (23.1%) were the most frequent injuries; and chest (55.6%), head (41.9%), and lower extremities (27.5%) were the most commonly associated injuries. Mechanical ventilation was required in 89.6%, emergency surgery was performed in 45% and vasopressors were used in 46.6% of patients. The most commonly performed interventions during the ICU stay were surgery (39.5%) and chest tube insertion (33.3%). Of the 702 patients with abdomino-pelvic trauma, 115 (16.4%) patients did not survive. Associated head trauma and retroperitoneal hematoma, higher level of lactic acid on admission and ISS, and advanced age were independent risk factors for fatality. Compared with survivors, nonsurvivors were older in age; had higher ISS, ApACHE II, BMI, lactic acid, INR and creatinine, and lower GCS, PaO_2_/ FiO_2 _and platelet count; were more likely to be mechanically ventilated and on vasopressors; and were associated with more head injuries.

## Conclusion

In KSA, abdomino-pelvic traumas are serious injuries affecting mainly young male victims; however, with a lower mortality than predicted.

